# Molecular changes, histopathology, and ultrasonic vocalization acoustic profiles of systemically dehydrated rats

**DOI:** 10.1371/journal.pone.0322187

**Published:** 2025-04-22

**Authors:** Brooke Rodgers, Naila Cannes do Nascimento, Abigail Cox, Taylor W. Bailey, M. Preeti Sivasankar, Allison J. Schaser

**Affiliations:** 1 Department of Speech, Language, and Hearing Sciences, Purdue University, West Lafayette, Indiana, United States of America; 2 Department of Comparative Pathobiology, Purdue University, West Lafayette, Indiana, United States of America; University Hospital Eriangen at Friedrich-Alexander-University Erlangen-Numberg, GERMANY

## Abstract

Systemic hydration is known to promote optimal functioning of bodily systems—including the vocal folds. The impact of systemic dehydration on the biology of the vocal folds and the downstream effects of dehydration on voice output are not well understood. An *in vivo* rat model of systemic dehydration was employed to investigate vocal fold gene expression, histological changes, and acoustic changes in vocalization. Ultrasonic vocalizations (USVs) were recorded every day for 5 days (baseline), in male and female Long-Evans rats (N = 36, ages: 3–4 months) using an anticipatory reward paradigm. Next, rats were dehydrated (N = 18) using a published water-restriction model for 5 days or euhydrated (N = 18) and provided *ad libitum* access to water for 5 days. USVs were recorded daily during the dehydration/euhydration period. The USV variables were averaged at baseline and following dehydration/euhydration for individual animals, and the difference between these time periods was used for statistical analysis. USV analysis included total USV count, complexity ratio, duration (s), frequency range (kHz), and maximum intensity (dB). At the end of dehydration/euhydration, animals were euthanized, and kidney and vocal fold tissue samples were dissected and processed for histology and gene expression analysis. Compared to euhydrated rats, dehydrated male and female rats had significantly up-regulated gene expression of kidney renin (male *p* = 0.047; female *p* = 0.018), indicating physiologic dehydration. There were no statistically significant differences in the USV acoustic profile or histopathology between the two groups. Differential expression (*p* < 0.05) of several genes related to extracellular matrix remodeling, inflammatory responses, and water ion transport in the vocal folds was present. Our results indicate that mild systemic dehydration impacts gene expression in the vocal fold mucosa; however, these gene expression changes are not evident in the acoustic profile of vocalizations.

## Introduction

Water is integral for virtually all bodily functions and systems, and a lack of sufficient systemic hydration is associated with adverse effects on processes such as muscle function, thermoregulation, and cognition [1–7]. In 2005, the Institute of Medicine stated that adult males and females should consume 3.0 L and 2.1 L of fluids daily, respectively, to maintain a state of euhydration, or optimal hydration [7]. However, numerous studies suggest that the majority of people are likely not meeting daily recommendations of water consumption [1,8–10].

The role of hydration is especially important in the function of soft tissues in the body, such as the vocal folds. The vocal folds are bilateral multilayered, viscoelastic structures comprising the thyroarytenoid muscle, lamina propria, stratified squamous epithelium, and extracellular matrix (ECM). The thyroarytenoid muscle and lower layers of the lamina propria form the internal postural base of the vocal folds. The upper portion of the lamina propria, epithelial layer, and ECM form the vocal fold mucosa. The mucosa contains a high density of vibration-resistant collagen and elastin fibers and is lubricated by a layer of secretions [11]. The ECM contains proteoglycans and glycosaminoglycans (GAGs) that modulate tissue viscosity, wound repair, and fibrous structure [12–14]. The biomechanic properties of the vocal folds are influenced by systemic hydration levels, or the amount of water transported in the blood and distributed across the body [2,15–17].

Ethical considerations limit the types of dehydration studies that can be conducted in humans. As such, research about systemic dehydration in humans has been limited to using mild short-term models of systemic dehydration and subjective outcome measures of voice production. Studies have been conducted with a wide variety of participants with differing sex, age, occupational voice use, and level of voice training [2,18]. Across all groups, increasing systemic hydration has been shown to decrease phonation threshold pressure, which is associated with reduced vocal effort [19–22]. There is also evidence that increased systemic hydration prevents the onset of vocal fatigue and is a helpful treatment for phonotrauma [23,24]. Studies using imaging methods to investigate the vocal folds’ function have also shown that systemic dehydration causes negative changes to the vibratory patterns and incomplete closure of the vocal folds during phonation [19,21,25]. Few human studies have reported the effects of systemic dehydration on objective voice acoustic profile measures, and the results are inconsistent across participants. Yet, it appears that systemic dehydration may adversely impact perturbation measures of the voice [26]. The consensus of these human trials has been that ample hydration may help prevent negative effects to the voice following vocal challenges like fatigue, increased effort to maintain a consistent voice, and prolonged recovery after phonotraumas. However, the hydration status of the participants at baseline is not addressed consistently, and objective markers of dehydration are rarely reported in these studies. As a result, the conclusions from human studies of systemic dehydration and voice use must be evaluated critically.

Human studies are especially helpful in investigating the greater impacts of systemic dehydration on voice behavior and guiding clinical practice, but these studies are limited and therefore less informative about specific changes to underlying voice physiology. *Ex vivo* studies using animal tissues can be informative when studying dehydration effects on specific organs, like the vocal folds. A study using excised canine vocal fold tissue showed that when under systemic dehydration conditions, the vocal fold mucosa loses water faster than the deeper layers of the vocal folds [15]. Systemic dehydration has also been shown to alter the stress-strain relationship of soft tissues, like the vocal folds. A lack of hydration causes collagen and elastin fibers in the vocal folds to become stiffer and increases the relaxation time after stretching [27]. In human vocalization, the results from these studies translate to systemic dehydration interrupting the symmetric, biphasic movement of the vocal folds, resulting in a change to the sound produced and increased effort from the speaker to maintain a consistent voice. However, these *ex vivo* studies commonly use models of severe systemic dehydration (20–70% total mass loss) that are unrealistic in living systems. Additionally, *ex vivo* models of systemic dehydration cannot represent the multi-system dynamics involved in systemic hydration. Still, these studies provide important information about the specific changes made to the soft tissues of the vocal folds induced by systemic dehydration.

*In vivo* animal studies allow for more controlled models of systemic dehydration than human studies, and unlike *ex vivo* animal models, can represent the behavioral and multisystem interactions impacted by systemic dehydration. Previous *in vivo* animal studies of systemic dehydration have used imaging methods, histology, transcriptomics, and proteomics to investigate the impact of acute and chronic systemic dehydration on the vocal folds [28–35]. Proton density weighted magnetic resonance imaging (MRI) was used in a rat model of acute systemic dehydration to show changes in the total volume of water within the vocal folds [30,31]. The MRI images revealed that the vocal folds are sensitive to moderate and marked changes in hydration levels in the body and systemic dehydration causes a net loss of water in the vocal fold tissue. Proteomic and histological changes to the vocal fold tissue have been shown in rabbit and rat models of systemic dehydration [28,29,32–35]. In studies using male rabbits, RNA sequencing and global proteomic analysis were used to reveal molecular changes in the vocal folds caused by systemic dehydration [29,32,33]. Several proteins and RNA transcripts related to the composition of the vocal fold mucosa and ECM, ion transport, and inflammatory responses were found to be affected by systemic dehydration. Despite analyzing the complete vocal fold tissue in these studies, proteins and transcripts related to the thyroarytenoid muscle were not affected by systemic dehydration [29,32,33]. Changes to proteins related to the thyroarytenoid muscle were only observed in the vocal folds of rabbits that had been exposed to low-humidity conditions [36].

Following the results of the systemic dehydration studies in rabbits, changes to protein and gene expression related to the vocal fold mucosa and ECM were observed in a study of systemic dehydration using male rats [28]. In this study, systemic dehydration caused significant downregulation of interleukin-1α (*IL1α*) and desmoglein-1 (*DSG1*), and upregulation of hyaluronidase-2 (*Hyal2*) [28]. Interleukin genes regulate a group of cytokines secreted by white blood cells, suggesting that systemic dehydration impacts the inflammatory response in the vocal fold tissue [28,29]. Desmoglein genes regulate a group of transmembrane proteins that are important for maintaining cell-to-cell adhesion in the outer epithelial layer of the vocal folds [37]. Changes to desmoglein expression caused by systemic dehydration would impact the integrity of the vocal fold epithelium and may contribute to increased fatigue and phonotrauma incidence. Finally, hyaluronidase genes regulate the expression of enzymes that break down hyaluronan, the most common GAG present in the vocal fold mucosa. Hyaluronan binds to water and contributes to the viscosity of the vocal fold mucosa and is thought to influence phonation threshold pressure of the voice [12,35,38]. Systemic dehydration appears to lead to a reduction in the amount of hyaluronan in the vocal fold mucosa, which may contribute to increased vocal effort associated with dehydration.

The results of these *in vivo* animal studies indicate that systemic dehydration causes changes in vocal fold gene expression that leads to restructuring of the mucosa and ECM. Changes to the composition of the vocal fold mucosa and ECM could impact the vibratory properties of the vocal folds and the configuration of the glottis during phonation, leading to voice changes following vocal challenges including fatigue, vocal effort, and recovery, all of which are associated with systemic dehydration. However, these studies did not include measures of voice function with systemic dehydration. Additionally, gene and protein expression changes have not been studied in female rats or rabbits. There is a need to further investigate how systemic dehydration impacts the molecular composition, morphology, and physiology of the vocal folds to better understand why systemic dehydration is associated with voice changes in both sexes.

The majority of *in vivo* animal studies of systemic dehydration utilize acute models of dehydration, where diuretics or water withholding protocols are used to induce a state of systemic dehydration in a short period of time. Recently, water restriction has been employed by our group as a paradigm to model mild, systemic dehydration that more accurately reflects the reduced daily water consumption of the average human [28]. Under the water restriction protocol, animals are given a limited volume of water, each day, over multiple days, to induce physiologically relevant systemic dehydration. This model was used to confirm changes to the vocal fold mucosa at the gene expression level previously shown in a water withholding model of systemic dehydration in rats [28].

In this study, male and female rats were used to study the effects of systemic dehydration on the molecular composition and function of the vocal folds. Similar to humans, rat USVs are produced in the larynx and are modulated through motor patterns that control the thyroarytenoid and cricothyroid muscles [39–41]. However, unlike other mammals that produce sound through flow-induced vocal fold oscillations, rodent communicative USVs are aerodynamic whistle tones [39–43]. During whistle tone USVs, the thyroarytenoid muscle controls vocal fold adduction and the cricothyroid muscle controls the length of the vocal folds to manipulate the size and shape of the glottis [39,42,43]. Instead of vocal fold oscillations, sound is produced through complex air pattens formed by an airstream exiting the glottis and impinging upon other surfaces within the larynx [42–44]. The acoustic features of USVs are influenced by the intrinsic laryngeal muscle activity shaping the glottis, position of vocal tract structures, and subglottal pressure supply, rather than complex vibratory patterns of the vocal folds. Though the vocal folds of rats do not make complete contact during vocalization, the glottis shape formed by the adducted vocal folds influences the acoustic features of the USVs produced [44]. Previous studies have demonstrated that systemic dehydration primarily causes changes to the structure of the vocal fold mucosa. We hypothesize that structural changes to the vocal fold mucosa also have the potential to alter the shape of the glottis, resulting in changes to USVs acoustic parameters.

The present study aims to address the gap in the literature of systemic dehydration and vocalization by using an *in vivo* rat model of mild systemic dehydration. Many studies investigating systemic dehydration have shown molecular changes to the vocal fold mucosa or subjective perceptual and behavioral voice changes separately, but few studies have investigated these changes simultaneously. Additionally, very few studies include objective acoustic analysis of the voice after dehydration. This is the first study to combine examination of vocal fold molecular and histopathological changes with changes to the acoustic profile of vocalizations produced by rats undergoing systemic dehydration. Our results begin to explore the impact of gene expression changes on objective voice measures.

## Methods

### Animals

The procedures included in this study were performed in accordance with a protocol approved by the Purdue University Animal Care and Use Committee (PACUC 2111002220, approved April 2020). Thirty-six 3-month-old Long-Evans rats — 18 males (pre-dehydration weight 263-414g) and 18 females (pre-dehydration weight 197-250g) — acquired from Envigo (Indianapolis, USA) were used in the study. The rats were housed in individual cages with enrichment toys in a temperature and humidity-controlled animal facility under a 12hr light/12hr dark cycle. The animals were housed individually to allow the researchers to control their water intake. Though the rats were housed individually, they had visual and olfactory contact with the other rats in the study. After arrival, the rats acclimated to the housing conditions for one week before beginning experiments. During this time, the rats underwent daily socialization through human interaction to reduce stress and facilitate handling during the behavioral portion of the study. All the rats were given *ad libitum* access to food (Teklad 2018S Rodent Diet by Inotiv, West Lafayette, IN, USA) and water during the acclimation period. This study did not include any painful survival procedures, and no anesthetics nor analgesics were used. All animals were euthanized using CO_2_ at the end of the study before tissue collection.

### Dehydration protocol

**Fig 1:**
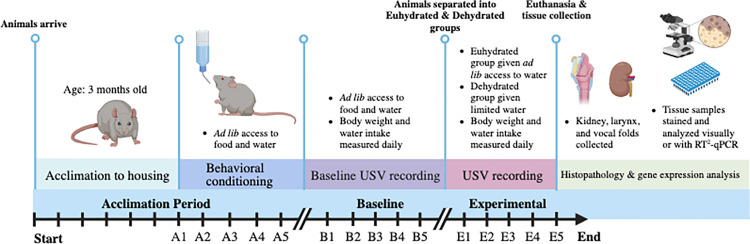
Experimental timeline. Schematic of the timeline used for the acclimation period (A1-A5), the behavioral conditioning period (B1-B5) and the experimental period (E1-E5). Information regarding the dehydration protocol, ultrasonic vocalization (USV) recording, and tissue collection is listed in each period. Image created with BioRender.com.

Mild systemic dehydration was induced in the animals using a validated five-day physiologically realistic water restriction protocol based on the baseline bodyweight of each individual animal [28]. A schematic showing the timeline of the dehydration protocol and data collection is shown in Fig 1. The daily water intake of each rat was determined by measuring the change in the volume of water inside the water bottle in each rat cage for each day of the five-day baseline recording period and adjusting for evaporation loss. The rats were also weighed each day of the baseline recording period. The average daily water intake and final bodyweight were recorded as baseline measurements.

After the baseline period, the rats were randomly divided into two groups for the five-day experimental period. The euhydration group (female n = 9 and male n = 9, total N = 18) continued to receive *ad libitum* access to food and water for the five-day experimental period. The dehydration group (female n = 9 and male n = 9, total N = 18) had restricted volume of water for the five-day experimental period. To induce systemic dehydration, rats in the dehydration group were given 4 mL of water per 100 g of bodyweight measured at baseline. All animals were weighed and USVs were recorded on each day of the experimental period using the same procedure as the baseline recordings. After the fifth day of the experimental period, all the rats from both groups were euthanized using CO_2_ and vocal fold and kidney tissue were collected.

### Ultrasonic vocalizations (USVs)

#### Acclimation and behavioral conditioning.

Following the initial acclimation period to housing conditions, the animals were prepared for the study with a 5-day behavioral conditioning period. Animals were trained to vocalize while inside of a recording chamber (SmartChamber by Metris, Hoofddorp, Netherlands) in anticipation of a food reward using an adapted anticipatory reward paradigm [45,46]. Under the anticipatory reward elicitation paradigm the animals are allowed to vocalize freely, capturing the animals’ innate vocalizations. During behavioral conditioning, each rat was removed from its home cage and placed into a clean, empty cage inside a closed recording chamber for a period of ten minutes. Immediately upon removal from the recording chambers, the rats were given a food reward — either fruit loops or veggie straws — in their home cage. The food rewards were provided on a fixed-interval reinforcement schedule at the end of each 10-minute session, and the treat type was alternated each day to increase salience and interest in the reward. Fruit loops and veggie straws were chosen for low moisture content. and to alternate between sweet and salty rewards. Over the 5-day conditioning period, the rats built an association with being inside the recording chamber and receiving the food reward and learned to produce USVs while inside the chamber in anticipation of leaving the chamber and receiving the reward [45,46]. During the behavioral conditioning period, trial recordings were made to ensure the rats were producing vocalizations during the conditioning sessions. Any animals that did not produce vocalizations during the conditioning sessions were excluded from the study (N=0). All rats were given *ad libitum* access to food and water during the behavioral conditioning period.

#### USV recordings.

Following the behavioral conditioning period, USVs were recorded for 10 consecutive days at the same time of day using the Sonotrack (Metris) recording software package and SmartChamber (Metris) system. Up to four animals were recorded during a single 10-minute session using four separate SmartChambers. Each SmartChamber is enclosed in a Faraday cage and lined with anechoic foam to remove the possibility of crosstalk between chambers. A wide frequency range microphone located at the top of each recording chamber was used to record the USVs produced by each rat. The recording procedures were identical to the behavioral conditioning period, other than the microphone being active. After the 10-minute recording period, the rats were removed from the recording chambers, returned to the home cage, and immediately given a food reward. The animals were recorded in a random order and placed in different recording chambers each day. The baseline period included the first five days of recording, where all animals received water *ad libitum*. The experimental period (dehydration/euhydration) included the second five days of recording. All rats received unrestricted access to food during the entire study.

#### USV statistical analyses.

The USVs were recorded and analyzed using the Sonotrack (Metris) software package, which identifies and classifies USVs based on their acoustic characteristics. For our purposes, only USVs in the 50 kHz range that were longer than 10 ms were included for analysis. USVs shorter than 10 ms were excluded from analysis to reduce the amount of noise incorrectly identified as vocalizations by the identification software, following information gained from previous unpublished USV collection by our group. USVs were separated into two broad classification groups — simple or complex. The classification parameters were adapted from previously published protocols and the Metris USV categorization guidelines [47]. USVs with only one acoustic element (flat, up/down, and U-shaped USVs) were considered simple vocalizations, and USVs with multiple acoustic elements (trailing, step up/down, and complex USVs) were considered complex vocalizations for analysis. The USV characteristics of interest were the total USV count, complexity ratio (calculated as the number of simple USVs divided by the number of complex USVs), duration (s), frequency range (kHz) (calculated as Fmax – Fmin), and the maximum intensity (dB).

The total number of USVs produced was summed separately for the baseline and experimental periods for each animal, then the difference between the baseline and experimental periods was calculated and normalized to the baseline value for statistical analysis. The complexity ratio was calculated for each individual rat on every recording day, then averaged separately for the baseline and experimental periods. The difference in the average complexity ratio from baseline to the experimental period was used for statistical analysis. The other three variables (USV duration, frequency range, and maximum intensity) were averaged over all recording sessions in each period, then the difference between the averaged values for the baseline and experimental periods was calculated and normalized to the baseline value for statistical analyses. Estrous cycle was not measured in the female rats, but the 4–5 day cycles of Long-Evans rats were captured within both the baseline and experimental periods [35,48–50]. We did not control for estrous stage in our statistical analyses, but instead averaged over each period.

Besides complexity ratio, statistical analyses were stratified between simple and complex USVs for all variables due to the classification parameters. Two-way analysis of variance (ANOVA) models were performed with two, 2-level factors (sex and hydration group) and an outcome variable of the normalized difference in each acoustic variable from baseline to the experimental period. Differences in the USV characteristics were considered statistically significant when *p* ≤ 0.05.

### Gene expression quantitation

#### Total RNA extraction.

Kidney and larynx tissue samples were collected from all animals following euthanasia. The vocal fold mucosa from 20 rats (n=5/group) was microdissected for gene expression. The remaining larynges were used for histological analysis described below. Vocal fold and kidney samples were stored in RNAlater® Stabilization Solution (Invitrogen™ by Thermo Fisher Scientific, Waltham, MA, USA) at -20°C before RNA extraction. Total RNA from vocal fold and kidney samples was extracted using the RNeasy Fibrous Tissue Mini Kit, including on-column DNAse I digestion step, and the RNeasy Plus Mini Kit (QIAGEN, Hilden, Germany) following the manufacturer’s protocol, respectively. Spectrophotometry (NanoDrop™, Thermo Fisher Scientific) was used to assess the RNA concentration and quality.

#### cDNA synthesis and RT^2^-qPCR array.

The cDNA synthesis of vocal fold tissue was performed with 220 ng of total RNA using the RT^2^ First Strand Kit (QIAGEN). To evaluate gene expression in the vocal folds, a custom RT² Profiler PCR Array (QIAGEN) comprising 91 genes related to extracellular matrix remodeling and regulation was tested for each sample (n = 5/group). The 96-well plate included two normalizer genes (hypoxanthine phosphoribosyl-transferase 1: *Hprt1* and β-actin: *Actb*), a positive control, a genomic DNA control as a negative amplification control, and a reverse-transcription control. The primers’ sequences of the RT² Profiler PCR Array are proprietary. A complete list of genes and plate layout are provided in S1 Table. The genes included in the array have functions related to the composition of the vocal fold epithelium, ECM remodeling, and inflammatory responses and were chosen based on previous studies [28,29,33,34]. The results of these previous studies indicated that systemic dehydration causes changes to the molecular composition of the vocal fold mucosa. We hypothesize that structural changes to the vocal fold mucosa associated with systemic dehydration impact the biomechanics of phonation and the glottal configuration, and may cause changes in rodent USV acoustic measures.

For kidney samples, the cDNA synthesis was prepared with 1.0 mg of total RNA using SuperScript™ IV VILO™ Master Mix (Invitrogen™ by Thermo Fisher Scientific). The primers’ sequences used for renin (*Ren*) and *Actb* are described in a previous study [51]. The qPCR assays for vocal fold and kidney were carried out in a QuantStudio™ 3 Real-Time PCR System (Applied Biosystems™) with the following thermal cycling conditions: 95 °C for 10 min; 40 cycles of 95 °C for 15 sec, and 60 °C for 1 min; followed by melt curve stage of 95 °C for 15 sec, 60 °C for 1 min, 95 °C for 1 sec. The 2^−ΔΔCT^ method [52] was applied to analyze the relative gene expression in the vocal fold tissue and kidney using *Hprt1* and *Actb* combined, or *Actb* only as normalizers, respectively.

### Histological analysis and hyaluronan quantification

Larynges excised from 16 rats (n = 4/group) were immediately fixed in 10% neutral buffered formalin, processed, and stained with hematoxylin and eosin (HE) or pentachrome according to standard procedures. All microscope slides underwent histological evaluation by a veterinary pathologist and were evaluated based on standard tissue morphology.

Each larynx was also sectioned for vocal fold hyaluronan quantification by staining serially sectioned larynges with Alcian blue (pH 2.5) with or without pre-incubation with 0.5 mg/ml hyaluronidase (MilliporeSigma, St. Louis, MO) [12]. Alcian blue stains glycosaminoglycans (GAGs), and hyaluronan is the most common GAG in the vocal fold mucosa. GAGs are highly polar molecules that easily bind to water, and are used in the body as lubrication that contributes to the viscosity of the vocal fold mucosa [11]. In previous studies, we observed changes in the expression of genes regulating hyaluronan and the total hyaluronan content of the vocal fold mucosa [28,35]. We hypothesized that systemic dehydration may alter the GAG content of the vocal fold tissue, also changing the viscosity of the vocal folds and glottal configuration during vocalization. The hyaluronan in the vocal folds was quantified by determining the difference in positive Alcian blue stain in pre- and post-hyaluronidase treated slides. Slides were analyzed by a veterinary pathologist and quantified by digital image analysis using an Aperio Versa® slide scanner (Aperio Technologies, Vista, CA) to digitize the slides and ImageScope software (v.12.3.3.5048) to quantify the positive signal. Specifically, an algorithm quantifying positive Alcian blue stain in the vocal fold tissue quantified the percent difference in Alcian blue stain between pre- and post-hyaluronidase Alcian blue stained slides.

#### Gene expression and hyaluronan quantification statistical analysis.

The relative gene expression of the vocal fold target genes and kidney *Ren* were independently compared between euhydrated and dehydrated groups for both sexes. The relative expression data were tested for normal distribution with the Shapiro-Wilk test (a= 0.05); unpaired t-test or Mann-Whitney test were applied, when data passed normality or did not pass normality, respectively. For data with unequal variances, Welch’s correction was used. Genes were considered differentially expressed between euhydrated and dehydrated males or females when *p* ≤ 0.05. The same process was used to compare the hyaluronan content in the vocal folds between euhydrated and dehydrated rats of both sexes. For the RT^2^-qPCR Array analysis, *p*-values were adjusted using the Benjamini and Hochberg step-up procedure to accommodate multiple comparisons and control for false discovery rate (FDR).

### Statistical analyses and data availability

All statistical analyses described above were performed using R Statistical Software (v4.2.3; R Core Team 2023, Vienna, Austria). All files for this study including USV recordings and acoustic analysis, qPCR results, biometric data, and histology images can be found on the Purdue University Research Repository (https://purr.purdue.edu/publications/4526/1; https://doi.org/10.4231/SXCE-9X65).

## Results

The male and female rats in the euhydrated group gained an average of 3.2% and 1.3% of their baseline bodyweight, respectively, by the end of the study. This increase in bodyweight was expected as part of the natural aging process resulting from the young-adult age of the animals at the start of the baseline period. The male and female rats in the dehydration group had an average maximum bodyweight loss of 0.2% and 1.7%, respectively, indicative of mild systemic dehydration.

### Renin gene expression

**Fig 2:**
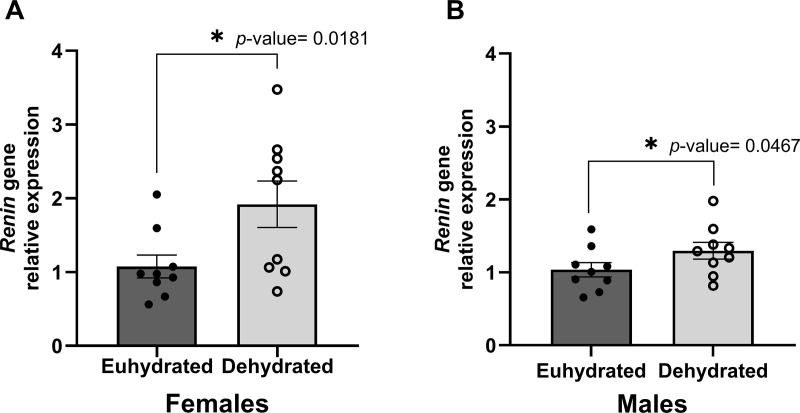
Dehydrated animals had increased kidney renin expression. Plots showing the relative expression of renin gene (*Ren*) in the kidneys of female (A) and male (B) rats. The relative *Ren* gene expression was normalized to β-actin and compared to the euhydrated groups (reference groups). For both sexes, dehydrated animals had significantly greater expression of renin. Error bars represent the standard error of the mean value.

The expression of renin (*Ren*) gene in the kidney was significantly increased in the dehydrated male (*p* = 0.0467) and female rats (*p* = 0.0181) compared to the euhydrated control groups by 1.3-fold and 1.8-fold, respectively (Fig 2). Increased renin expression is an indicator of systemic dehydration.

### USVs

Over 30,000 vocalizations were identified by the USV identification software and included for statistical analysis. Of the USVs identified, 5,982 were classified as complex vocalizations and 24,200 were classified as simple vocalizations.

**Fig 3:**
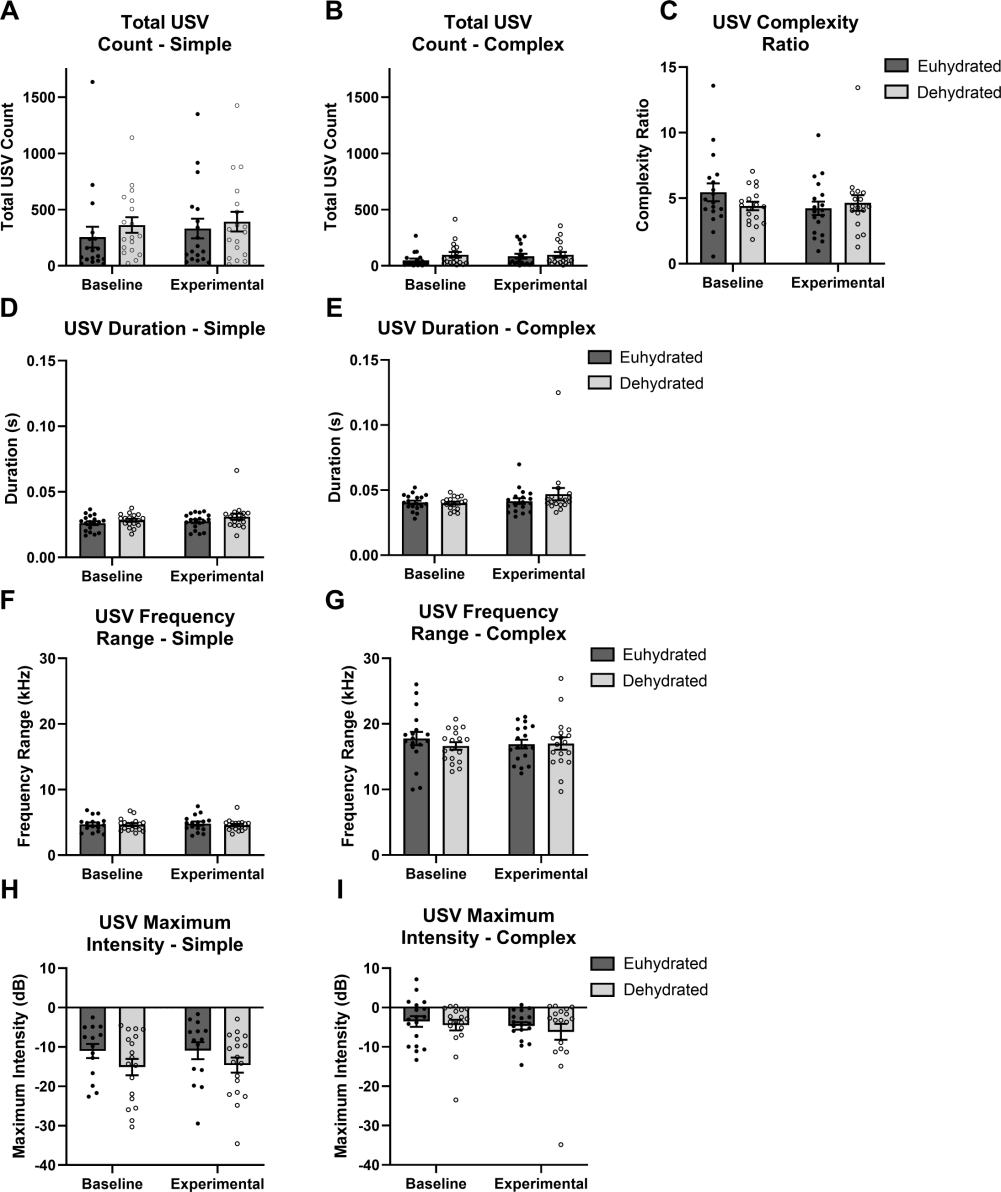
No differences were observed in the USV group averages. Group averages (including males and females) for the USV characteristics shown for each hydration group (euhydrated and dehydrated) and time point (baseline and experimental period). Simple (left panel) and complex (right panel) USVs were analyzed and shown separately for total USV count (A, B), duration (D, E), frequency range (F, G), and maximum intensity (H, I). C shows the group averages for the complexity ratio, which includes both simple and complex USVs. The error bars represent the standard error of the mean value.

There were no statistically significant effects of hydration group observed in any of the acoustic variables of interest for either vocalization type: total USV count (simple: *p* = 0.191; complex: *p* = 0.119), complexity ratio (*p* = 0.465), USV duration (simple: *p* = 0.705; complex: *p* = 0.292), USV frequency range (simple: *p* = 0.370; complex: *p* = 0.661), or USV intensity (simple: *p* = 0.722; complex: *p* = 0.913). The group averages of the differences between baseline and the experimental period for each variable are shown in Table 1. Plots of the group averages for each time period are shown in Fig 3. There was a statistically significant main effect of sex (*p* = 0.030) on USV complexity ratio, but no effect due to hydration group. Compared to the baseline value, female rats had an average increase of 25.1% while male rats had an average decrease of 19.7% in complexity ratio. This indicates that female rats produced a larger portion of simple USVs and male rats produced a larger portion of complex USVs during the experimental period compared to baseline. These differences were not large enough to also cause a significant difference in the total number of simple and complex vocalizations produced, though.

**Table 1:** Group average changes in USV characteristics compared to baseline.

	Euhydrated Rats		Dehydrated Rats	
	Simple USVs	Complex USVs	Simple USVs	Complex USVs
	Difference (% change)	Difference (% change)	Difference (% change)	Difference (% change)
**Average Total Count**	76.2 (+78.2%)	35.9 (+109%)	30.4 (+21.0%)	0.222 (+30.8%)
**Average Complexity Ratio (simple/complex)**	−1.226 (-4.60%)		0.236 (9.98%)	
**Average Duration (s)**	0.00146 (+6.80%)	0.00107 (+4.06%)	0.00279 (+9.86%)	0.00686 (+16.7%)
**Average Frequency Range (kHz)**	0.0929 (+3.04%)	−0.857 (-0.063%)	−0.1768 (-2.36%)	0.384 (+3.91%)
**Average Maximum Intensity (dB)**	0.0637 (+11.7%)	−1.14 (+85.9%)	−0.564 (+21.3%)	−1.71 (+107%)

Group averages for each USV characteristic calculated as the difference between the baseline and experimental periods (Difference = [Experimental – Baseline] and the percent change from the baseline value (%Change = Difference/Baseline x 100). The sign of the average value indicates an increase (+) or decrease (−) in the average value during the experimental period compared to baseline.

All animals produced more simple than complex USVs at both time points, resulting in average complexity ratios greater than 1. Complex USVs always had greater durations, frequency ranges, and maximum intensities than simple USVs (Fig 3). All animals experienced small increases in the total number and duration of simple and complex USVs produced during the experimental period. Animals in the euhydrated group showed a slight decrease in the average USV complexity ratio during the experimental period (dehydration/euhydration), indicating that of the total USVs produced, more complex vocalizations or fewer simple vocalizations were made compared to the baseline period. However, this change was not statistically significant compared to the dehydration group. The animals in the euhydrated group showed an increase in the frequency range of simple USVs and a small decrease in the frequency range of complex USVs. Animals in the dehydrated group showed the opposite trend, where the frequency range of simple USVs decreased and the frequency range of complex USVs increased during the experimental period compared to baseline. The average changes to frequency were small (less than 4%), however, and did not result in a statistically significant effect due to hydration. All animals showed increased maximum intensity of all USVs during the experimental period compared to baseline. The percent change from baseline for the maximum intensity of complex USVs appears large for both groups, but the true difference in intensity was less than 2 dB. This seemingly large change was likely because the maximum intensity ranged from -10 dB to 1 dB for complex USVs and because of the large variability in intensity between vocalizations.

### Extracellular matrix remodeling and regulation-related gene expression in the vocal folds

**Fig 4:**
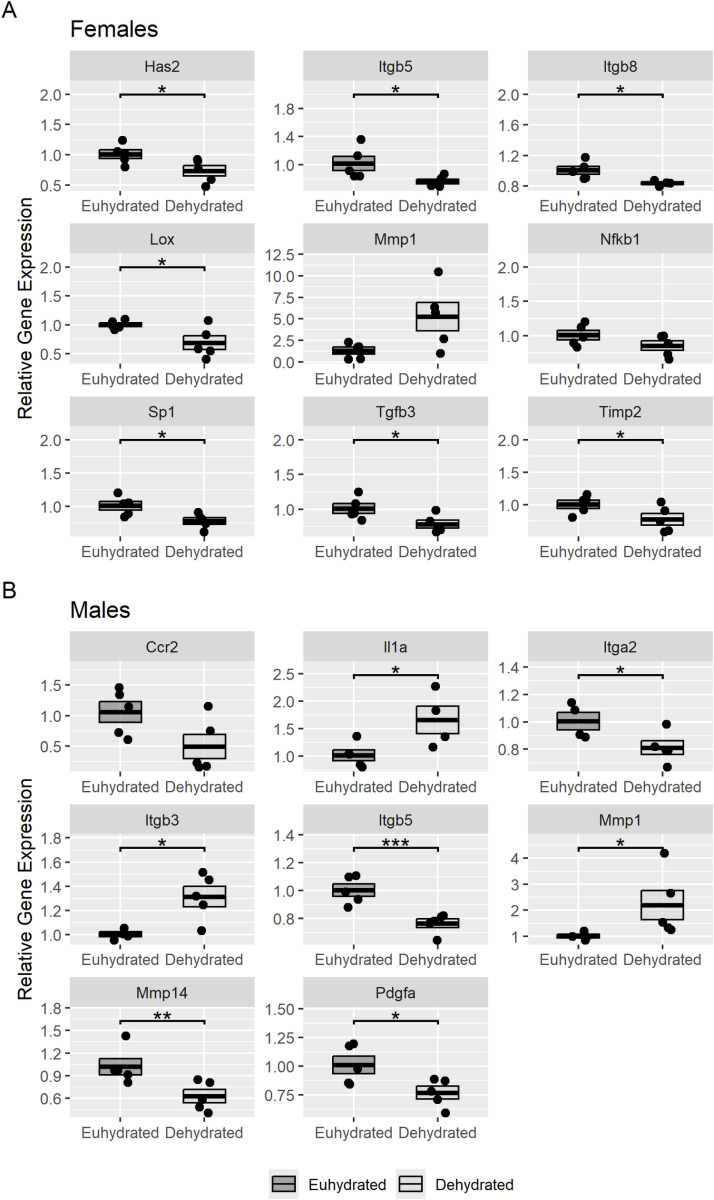
Differential gene expression in male and female dehydrated rats. Boxplots showing the relative gene expression in the vocal fold mucosa of female (A) and male (B) euhydrated and dehydrated rats. Genes that were significantly different between the hydration groups or close to significant are included (9 genes for females; 8 genes for males). Euhydrated groups are the reference and therefore have a relative quantification = 1.0. The y-axis range is scaled for each individual gene to aid visibility. The error bars represent the standard error of the mean. Asterisks indicate genes with relative expression differences that were statistically significant (unadjusted *p* < 0.05).

The results for the RT^2^-PCR array analysis comparing the gene expression in the vocal fold mucosa between the euhydrated and dehydrated groups are shown in Fig 4 and Table 2. Seven genes were significantly down-regulated in the vocal fold mucosa of dehydrated female rats compared to euhydrated females (Fig 4A), while four genes were down-regulated and three were up-regulated in the vocal fold mucosa of dehydrated male rats compared to euhydrated males (Fig 4B) (*p* < 0.05). A list of all the genes significantly altered by dehydration is shown in Table 2. After *p*-values were adjusted using the Benjamini and Hochberg procedure, only one gene (*Itgb5*, *p*-adjusted = 0.026) was found to have significantly altered expression between the male euhydrated and dehydrated groups.

**Table 2:** Differentially expressed genes in the vocal folds of dehydrated female and male rats.

Gene symbol	Gene name	Function	Fold Change	*P*-Value	Adjusted P-Value
**Females**					
*Has2*	Hyaluronan synthase 2	Hyaluronic acid synthesis	0.711	0.032	0.53
*Itgb5*	Integrin beta subunit 5	Cell adhesion & signal transduction	0.759	0.042	0.53
*Itgb8*	Integrin beta subunit 8	Cell adhesion & signal transduction	0.831	0.012	0.53
*Lox*	Lysyl oxidase	Assembly of ECM	0.651	0.048	0.53
*Mmp1*	Matrix metallopeptidase 1	Collagen degradation	4.02	0.058	0.53
*Nfkb1*	Nuclear factor kappa B subunit 1	Transcription factor related to inflammatory response	0.843	0.051	0.53
*Sp1*	Sp1 Transcription Factor	Zinc finger transcription factor	0.773	0.034	0.53
*Tgfb3*	Transforming growth factor beta 3	Cell differentiation and wound healing	0.776	0.038	0.53
*Timp2*	Tissue inhibitor of metalloproteinases 2	Suppresses proliferation of endothelial cells	0.757	0.029	0.53
**Males**					
*Ccr2*	C-C motif chemokine receptor 2	Regulates T-cell cytokine expression and differentiation	0.357	0.057	0.57
*Il1a*	Interleukin 1 alpha	Inflammatory response and apoptosis	1.61	0.043	0.51
*Itga2*	Integrin alpha subunit 2	Cell adhesion & signal transduction	0.786	0.018	0.28
*Itgb3*	Integrin beta subunit 3	Cell adhesion & signal transduction	1.31	0.018	0.28
*Itgb5*	Integrin beta subunit 5	Cell adhesion & signal transduction	0.763	0.00032	0.026
*Mmp1*	Matrix metallopeptidase 1	Collagen degradation	2.11	0.015	0.28
*Mmp14*	Matrix metallopeptidase 14	ECM breakdown	0.606	0.0056	0.23
*Pdgfa*	Platelet derived growth factor subunit A	Regulates cell proliferation	0.761	0.045	0.51

Table showing the gene symbols, gene names, function, and fold change in expression in the vocal fold mucosa of dehydrated male and female rats compared to the euhydrated group, and the *p*-value. Fold changes <1 indicate reduced expression and fold changes >1 indicate increased expression in the dehydrated animals compared to euhydrated. *P*-values were adjusted using the Benjamini and Hochberg procedure to control for false discovery rate. ECM is the abbreviation for extracellular matrix.

Of the genes included in the qPCR array, female dehydrated rats showed significantly reduced expression of hyaluronan synthase 2 (*Hyal2*, *p* = 0.032), some integrin subunits (*Itgb5, p* = 0.042; *Itgb8, p* = 0.012), lysyl oxidase (*Lox, p* = 0.048), specificity protein 1 transcription factor (*Sp1, p* = 0.034), a cytokine part of the transforming growth family (*Tgfb3, p* = 0.038), and a metalloproteinase inhibitor (*Timp2, p* = 0.029) in the vocal fold mucosa compared to the female rats in the euhydrated group. Of these genes, *Hyal2, Lox, Sp1,* and *Timp2* regulate different aspects of the extracellular matrix including hyaluronan content, cell differentiation, and matrix assembly [38,53–56]. The integrin subunit genes (*Itgb5* and *Itgb8*) contribute to the production of transmembrane receptors important for cell adhesion — impacting the integrity of the vocal fold epithelial layer [57]. Finally, *Tgfb3* regulates certain cytokines active during innate immunity and inflammatory response [58]. However, after adjusting for false discovery rate (FDR), there were no genes significantly altered between the female euhydrated and dehydrated groups.

Compared to male euhydrated rats, male dehydrated rats had significantly reduced expression of some integrin subunits (*Itga2, p* = 0.0318; *Itgb5, p* = 0.00032), one type of matrix metalloproteinase (*Mmp14, p* = 0.0056), and a platelet derived growth factor (*Pdgfa, p* = 0.045). Dehydrated male rats showed significantly increased expression of an interleukin (*Il1a, p* = 0.043), one integrin subunit (*Itgb3, p* = 0.018), and one type of matrix metalloproteinase (*Mmp1, p* = 0.015) compared to the euhydrated group. Matrix metalloproteinase genes break down structural proteins, including collagen, in the ECM and have been known to be part of inflammatory response [53,54]. Interleukins also play important roles in regulating innate immunity and inflammatory response, and are part of the apoptotic pathway [28,29]. *Pdgfa* regulates aspects of cell growth and differentiation, and it especially important in wound healing [59]. In male rats, systemic dehydration was shown to affect genes related to ECM regulation and remodeling, epithelial integrity, and innate immunity and inflammatory response in the vocal fold mucosa, similar to the female groups. However, after adjusting for FDR, expression of only one integrin subunit (*Itgb5, p*-adjusted = 0.026) was found to be significantly altered in the male groups.

Nuclear factor kappa B subunit 1 (*Nfkb1*), which is involved in immune responses, had a lower expression in dehydrated females (−1.2-fold) compared to euhydrated females, but this lower expression level was not statistically significant (*p* = 0.051). In dehydrated males, expression of C-C motif chemokine receptor 2 (*Ccr2*) gene — which encodes transmembrane receptor proteins that recruit immune cells — was decreased by −2.8-fold compared to euhydrated males; however, this decrease was not significant (*p* = 0.057).

Interestingly, the matrix metallopeptidase 1 (*Mmp1*) gene, which was significantly up-regulated in male dehydrated rats before adjusting for FDR, also showed a higher expression in dehydrated females compared to euhydrated (4-fold change); however, the difference was not statistically significant (*p* = 0.058). Unlike the males, systemic dehydration was shown to reduce expression of *Timp2* — an inhibitor of matrix metalloproteinases — in the female rats. Additionally, systemic dehydration caused reduced expression of some integrin subunits in both males and females, but dehydrated male rats also had increased expression of *Itgb3* compared to the euhydrated group. Of the integrin genes affected by systemic dehydration, only *Itgb5* was found to be differentially expressed in both males and females, and it was the only gene found significantly altered between the male groups after adjusting for FDR.

### Histological analysis and hyaluronan quantification

**Fig 5:**
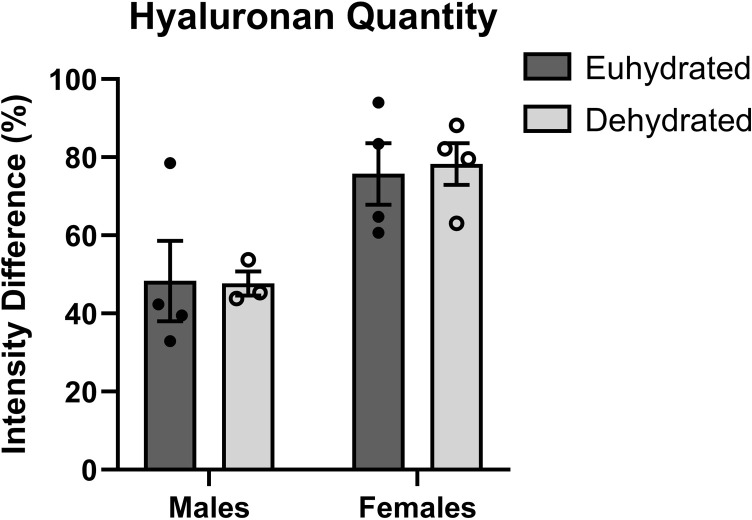
Results for vocal fold hyaluronidase quantification. Plot showing the change in relative Alcian blue intensity following hyaluronidase incubation for female (left panel) and male (right panel) rats in the euhydrated and dehydrated groups. Error bars represent the standard error of the mean value.

No differences in the vocal folds were observed for either sex or hydration group. The results of the vocal fold hyaluronan quantification are shown in Fig 5. Representative images of the pentachrome stained and Alcian blue treated vocal fold sections are provided in S2–S4 Figs. Male and female rats were observed to have different levels of vocal fold hyaluronan and were separated for statistical analysis. Results of the Mann-Whitney tests revealed no statistically significant differences between the euhydrated and dehydrated animals for males (*p* = 0.4) or females (*p* = 1).

## Discussion

In this study, we used a physiologically relevant model of systemic dehydration to investigate molecular and histological changes to the vocal folds as well as the resulting vocalization behavior in female and male rats. The results from this study support and expand upon the results of previous work from our group [28,29,32–35]. The rats in the dehydration group experienced significant reductions in bodyweight following the water restriction protocol — a change not observed in the rats given unrestricted access to water. At the end of the study, the dehydrated rats exhibited significantly increased renin gene expression in the kidney compared to the euhydrated rats, indicating that the water restriction protocol successfully induced mild systemic dehydration. Similar to our previous studies, we have shown that mild systemic dehydration causes gene expression changes at the level of the vocal fold mucosa [28,29,35], but these changes did not have a direct observable effect on the USV characteristics analyzed or hyaluronan content.

There were no significant differences between the two groups of rats, regardless of sex, in the total number of USVs produced, duration, frequency range, or maximum intensity of USVs. There was a significant effect due to sex observed in the USVs produced over the baseline and experimental periods, where male and female animals experienced different trends in the proportion of simple and complex USVs produced. The lack of differences in the vocalizations between the dehydrated and euhydrated groups indicates that mild systemic dehydration did not alter either the rats’ ability to vocalize in this elicitation paradigm or acoustic features of the USVs produced. The specific acoustic variables analyzed were chosen *a priori* to reflect clinical symptoms of systemic dehydration. It is possible that there were significant changes to other acoustic features not included in this study. Additionally, histological analysis of the vocal fold tissue revealed no significant differences between the euhydrated and dehydrated rats. No morphologic differences were observed in HE or pentachrome stained vocal fold slides between either hydration group. Furthermore, hyaluronan quantification revealed no differences in the amount of hyaluronan in the vocal folds between dehydrated and euhydrated rats.

In a previous study of moderate systemic dehydration, vocal fold sections from euhydrated and dehydrated female rats in different estrous stages were compared. Differences in vocal fold hyaluronan content between the dehydrated and euhydrated rats were present, but dehydration was not found to affect the overall morphology of the vocal fold tissue [35]. In the current study, the rats experienced mild systemic dehydration that did not result in significant loss of hyaluronan in the vocal fold tissue. We did not control for estrous stage in the female rats included in this study, which may contribute to the lack of observable differences in hyaluronan content between the female groups. However, there were no significant differences in hyaluronan content between the male groups either, further indicating that the mild level of systemic dehydration employed in this study may not impact hyaluronan content. Additionally, in the current study, the dehydrated rats were given a limited volume of water over several days to induce systemic dehydration rather than using water withholding to induce acute dehydration. Future work should investigate the physiologic differences in homeostatic response to systemic water withholding and water restriction on the composition of the vocal folds.

Despite the lack of change found in the USVs and histopathology, changes were observed in gene expression of the dehydrated rats’ vocal fold mucosa. Compared to the euhydrated rats, dehydrated rats had significantly altered expression of genes associated with the inflammatory response, epithelial integrity, and ECM regulation based on the unadjusted p-values. Male and female rats had different gene expression patterns and total hyaluronan content, suggesting that sex differences may modulate the response of the vocal fold tissue to systemic dehydration. Future work will further investigate the role of sex hormones in systemic dehydration induced changes to the vocal folds.

In the current study, systemic dehydration was shown to impact genes related to similar functions in the male and female rats, but the expression patterns were different. Only one gene was found to be significantly downregulated in both sexes — *Itgb5*. This was also the only gene found to be significantly altered in the male rats after controlling for FDR. Downregulation of *Itgb5* results in reduced expression of subunits of transmembrane proteins that have a role in cell adhesion, indicating that systemic dehydration has negative effects on vocal fold epithelial integrity [57]. Both male and female dehydrated rats also had comparatively increased expression of *Mmp1*, although the difference in expression was not significant in the female groups and not significant in the male groups after adjusting for FDR. *Mmp1* codes a group of enzymes that breakdown extracellular matrix proteins and are associated with inflammation [53,54]. Male dehydrated rats showed increased expression of *Il1a*. The increased *Il1a* gene expression seen in dehydrated males can contribute to *Mmp1* upregulation and can be indicative of cellular stress, likely associated with dehydration in this study [60–62]. Interestingly, the decreased expression of *Timp2* in the female could potentiate the impact of an increase in *Mmp1*.

The results of the gene expression analysis performed in this study support the findings of previous studies employing transcriptomics to study vocal fold systemic dehydration, but there are several apparent differences. A previous study using the same dehydration protocol with male Sprague-Dawley rats and analyzed the expression of genes related to membrane proteins, inflammatory cytokines, and ECM components [28]. Female dehydrated rats in our study had reduced expression of hyaluronan synthase (*Has2*) compared to the euhydrated group, which may have similar effects as upregulation of hyaluronidase [38]. Desmoglein was not included in our gene expression analysis, but both male and female dehydrated rats showed reduced expression of multiple integrins, which also contribute to cell-to-cell adhesion [37,57].

The gene expression, histological analysis, and USV results indicate that mild systemic dehydration may prime the vocal folds for functional deficits through changes in gene expression, but these changes do not meet the threshold to cause morphologic changes to the vocal folds or functional changes to vocalization in rats. The composition of the vocal fold tissue influences the shape of the glottis during USV production, but unlike in human vocalization, the vocal folds of rats do not make complete contact during USV production. Therefore, it is possible that changes to the vocal folds in rats may not affect the same acoustic features as in humans. As a result, subtle changes in the vocal fold mucosa resulting from changes in gene expression may not be well detected by the chosen acoustic variables. It is likely that acoustic features of rodent USVs would not change until dehydration becomes more severe. Our future work will continue to explore the utility of rodent USVs in translational studies and the best analysis practices.

The clinical implications of our findings are encouraging; however, we acknowledge some unavoidable limitations in our study. We used a validated 5-day dehydration paradigm to induce dehydration and used an anticipatory reward method to elicit vocalizations. Different methods of inducing dehydration and eliciting vocalizations (such as mating paradigms) will likely have different effects on the rats’ ability and motivation to produce USVs. Different USV elicitation methods utilize various social contexts to motivate animals to vocalize, and the context used influences the types of vocalizations produced [50]. The dehydration method used affected the vocal fold mucosa. However, the dehydration method employed in this study did not appear to alter the rats’ ability and motivation to produce USVs within the specific elicitation context used in this study.

The purpose of this study was to determine if mild systemic dehydration and changes to the vocal fold mucosa would alter USV acoustic characteristics. The results of this study indicate that gene expression changes in the vocal fold mucosa resulting from mild system dehydration do not alter the specific USV acoustic characteristics examined. Although rats have similar laryngeal anatomy to humans, the two species clearly differ in physiology as well as in the social functions of their vocalizations. For example, rats produce communicative USVs using whistle tones that are not used in human communication. Due to this difference in sound production, the mild systemic dehydration protocol employed may not have direct effects on the production of whistle tones but may result in subtle changes to vocal fold phonation in humans that are not captured in the USV model. It has been shown that rodents have greater activation of the intrinsic laryngeal muscles during USV production than other mammals during phonation related to the configurations required to produce whistle tones [39]. Based on the results of the current study, molecular and structural changes to the intrinsic muscles of the larynx may be a more relevant target when examining the effect of systemic dehydration on USV production. Future research should continue to study longer, and more severe models of suboptimal systemic hydration and the threshold of biochemical changes required to induce acoustic changes in vocalization measures in a variety of species.

Overall, the results presented above indicate that the voice is resistant to mild cases of systemic dehydration and can maintain function until the magnitude of systemic dehydration becomes more severe. We used a physiologically relevant model of systemic dehydration that mimics the vocal system of a human with suboptimal fluid intake. As mentioned previously, most people consume less than the recommended 64 fluid ounces of water per day and likely suffer from chronic, mild systemic dehydration [8–10]. Our results indicate that while mild cases of systemic dehydration may not cause stark acoustic differences to the voice or vocal fold composition, mild systemic dehydration does result in changes to the gene expression of the vocal fold mucosa and may leave the system vulnerable to additional factors, such as injury and overuse [2,18,21,26]. In this complex or challenge-based setting, underlying changes in gene expression, resulting from mild systemic dehydration, may result in changes to the acoustic features of the voice and may impact the functional use of the voice.

## Supporting Information

S1 Table All genes included in the custom RT^2^-PCR array for relative gene expression analysis. Table containing the gene symbols and gene descriptions for each gene included in the custom RT^2^-PCR array used to analyze the vocal fold mucosa tissue.
(PDF)

S2 Fig Representative images of male and female larynx coronal sections stained with pentachrome. Images of pentachrome stained coronal larynx slices. The top two panels show example images from female larynges, and the bottom two panels show images from male larynges. The panels on the left side represent images from the euhydrated group, and the panels on the right side represent images from the dehydrated group. The colors of the stain correspond to the following tissue types: black – nuclei and elastic fibers, yellow – collagen fibers, blue – mucins, bright red – fibrins, and red – muscle fibers.
(TIF)

S3 Fig Representative images of female vocal fold coronal sections stained with Alcian blue pre- and post-hyaluronidase incubation. Images of Alcian blue stained coronal larynx slices from female rats before and after hyaluronidase treatment. The top two panels show example images of larynx slices from a female rat in the euhydrated group, and the bottom two panels show images of larynx slices from a female rat in the dehydrated group. The panels on the right side show the larynx slices before hyaluronidase treatment, and the panels on the right show the same larynx slices after treatment.
(TIF)

S4 Fig Representative images of male vocal fold coronal sections stained with Alcian blue pre- and post-hyaluronidase incubation. Images of Alcian blue stained coronal larynx slices from male rats before and after hyaluronidase treatment. The top two panels show example images of larynx slices from a male rat in the euhydrated group, and the bottom two panels show images of larynx slices from a male rat in the dehydrated group. The panels on the right side show the larynx slices after staining, and the panels on the right show the same larynx slices after hyaluronidase treatment.
(TIF)
