# Supplementary material for: Molecular changes, histopathology, and ultrasonic vocalization acoustic profiles of systemically dehydrated rats
Source: PLoS One. 2025 Apr 22;20(4):e0322187. doi: 10.1371/journal.pone.0322187 (PMC12013907; doi:10.1371/journal.pone.0322187)
Supplement: S1 Table — Table containing the gene symbols and gene descriptions for each gene included in the custom RT2-PCR array used to analyze the vocal fold mucosa tissue. (PDF) [file pone.0322187.s001.pdf]

Gene symbol	Description
Hprt1	hypoxanthine phosphoribosyltransferase 1 [Source:RGD Symbol;Acc:2826]
Actb	actin, beta [Source:RGD Symbol;Acc:628837]
Has2	hyaluronan synthase 2 [Source:RGD Symbol;Acc:2781]
Hyal2	hyaluronidase 2 [Source:RGD Symbol;Acc:620321]
Fbln1	fibulin 1 [Source:RGD Symbol;Acc:1308667]
Fn1	fibronectin 1 [Source:RGD Symbol;Acc:2624]
Vcan	versican [Source:RGD Symbol;Acc:619940]
Vtn	vitronectin [Source:RGD Symbol;Acc:3967]
Efemp1	EGF containing fibulin extracellular matrix protein 1 [Source:RGD Symbol;Acc:1308528]
Acta2	actin alpha 2, smooth muscle [Source:RGD Symbol;Acc:621676]
Agt	angiotensinogen [Source:RGD Symbol;Acc:2069]
Ccl11	C-C motif chemokine ligand 11 [Source:RGD Symbol;Acc:3644]
Ccl12	C-C motif chemokine ligand 12 [Source:RGD Symbol;Acc:1309255]
Ccl3	C-C motif chemokine ligand 3 [Source:RGD Symbol;Acc:3647]
Ccn2	cellular communication network factor 2 [Source:RGD Symbol;Acc:621392]
Grem1	gremlin 1, DAN family BMP antagonist [Source:RGD Symbol;Acc:2359]
Il13	interleukin 13 [Source:RGD Symbol;Acc:68949]
Il13ra2	interleukin 13 receptor subunit alpha 2 [Source:RGD Symbol;Acc:620838]
Il4	interleukin 4 [Source:RGD Symbol;Acc:2898]
Il5	interleukin 5 [Source:RGD Symbol;Acc:2900]
Snai1	snail family transcriptional repressor 1 [Source:RGD Symbol;Acc:620758]
Bmp7	bone morphogenetic protein 7 [Source:RGD Symbol;Acc:620743]
Hgf	hepatocyte growth factor [Source:RGD Symbol;Acc:2794]
Ifng	interferon gamma [Source:RGD Symbol;Acc:2866]
Il10	interleukin 10 [Source:RGD Symbol;Acc:2886]
Col1a2	collagen type I alpha 2 chain [Source:RGD Symbol;Acc:621351]
Col3a1	collagen type III alpha 1 chain [Source:RGD Symbol;Acc:71029]
Lox	lysyl oxidase [Source:RGD Symbol;Acc:3015]
Mmp1	matrix metallopeptidase 1 [Source:RGD Symbol;Acc:1307917]
Mmp13	matrix metallopeptidase 13 [Source:RGD Symbol;Acc:620196]
Mmp14	matrix metallopeptidase 14 [Source:RGD Symbol;Acc:620198]
Mmp2	matrix metallopeptidase 2 [Source:RGD Symbol;Acc:621316]
Mmp3	matrix metallopeptidase 3 [Source:RGD Symbol;Acc:621317]
Mmp8	matrix metallopeptidase 8 [Source:RGD Symbol;Acc:631408]
Mmp9	matrix metallopeptidase 9 [Source:RGD Symbol;Acc:621320]
Plat	plasminogen activator, tissue type [Source:RGD Symbol;Acc:3342]
Plau	plasminogen activator, urokinase [Source:RGD Symbol;Acc:3343]
Plg	plasminogen [Source:RGD Symbol;Acc:619893]
Serpina1	serpin family A member 1 [Source:RGD Symbol;Acc:3326]
Serpine1	serpin family E member 1 [Source:RGD Symbol;Acc:3249]
Serpinh1	serpin family H member 1 [Source:RGD Symbol;Acc:69302]
Timp1	TIMP metallopeptidase inhibitor 1 [Source:RGD Symbol;Acc:621675]
Timp2	TIMP metallopeptidase inhibitor 2 [Source:RGD Symbol;Acc:61312]
Timp3	TIMP metallopeptidase inhibitor 3 [Source:RGD Symbol;Acc:3865]
Timp4	TIMP metallopeptidase inhibitor 4 [Source:RGD Symbol;Acc:69077]
Itga1	integrin subunit alpha 1 [Source:RGD Symbol;Acc:2923]

Itga2	integrin subunit alpha 2 [Source:RGD Symbol;Acc:621632]
Itga3	integrin subunit alpha 3 [Source:RGD Symbol;Acc:1310333]
Itgav	integrin subunit alpha V [Source:RGD Symbol;Acc:1310613]
Itgb1	integrin subunit beta 1 [Source:RGD Symbol;Acc:2927]
Itgb3	integrin subunit beta 3 [Source:RGD Symbol;Acc:628868]
Itgb5	integrin subunit beta 5 [Source:RGD Symbol;Acc:628869]
Itgb6	integrin subunit beta 6 [Source:RGD Symbol;Acc:1303119]
Itgb8	integrin subunit beta 8 [Source:RGD Symbol;Acc:1311374]
Ccr2	C-C motif chemokine receptor 2 [Source:RGD Symbol;Acc:620876]
Cxcr4	C-X-C motif chemokine receptor 4 [Source:RGD Symbol;Acc:620465]
Il1a	interleukin 1 alpha [Source:RGD Symbol;Acc:2890]
Il1b	interleukin 1 beta [Source:RGD Symbol;Acc:2891]
Ilk	integrin-linked kinase [Source:RGD Symbol;Acc:620063]
Tnf	tumor necrosis factor [Source:RGD Symbol;Acc:3876]
Edn1	endothelin 1 [Source:RGD Symbol;Acc:2532]
Egf	epidermal growth factor [Source:RGD Symbol;Acc:2542]
Pdgfa	platelet derived growth factor subunit A [Source:RGD Symbol;Acc:3282]
Pdgfb	platelet derived growth factor subunit B [Source:RGD Symbol;Acc:3283]
Vegfa	vascular endothelial growth factor A [Source:RGD Symbol;Acc:619991]
Cav1	caveolin 1 [Source:RGD Symbol;Acc:2280]
Dcn	decorin [Source:RGD Symbol;Acc:61895]
Eng	endoglin [Source:RGD Symbol;Acc:1593188]
Inhbe	inhibin subunit beta E [Source:RGD Symbol;Acc:621196]
Ltbp1	latent transforming growth factor beta binding protein 1 [Source:RGD Symbol;Acc:68379]
Smad2	SMAD family member 2 [Source:RGD Symbol;Acc:3031]
Smad3	SMAD family member 3 [Source:RGD Symbol;Acc:3032]
Smad4	SMAD family member 4 [Source:RGD Symbol;Acc:3033]
Smad6	SMAD family member 6 [Source:RGD Symbol;Acc:1305069]
Smad7	SMAD family member 7 [Source:RGD Symbol;Acc:69314]
Tgfb1	transforming growth factor, beta 1 [Source:RGD Symbol;Acc:69051]
Tgfb2	transforming growth factor, beta 2 [Source:RGD Symbol;Acc:70491]
Tgfb3	transforming growth factor, beta 3 [Source:RGD Symbol;Acc:3851]
Tgfbr1	transforming growth factor, beta receptor 1 [Source:RGD Symbol;Acc:3852]
Tgfbr2	transforming growth factor, beta receptor 2 [Source:RGD Symbol;Acc:69651]
Tgif1	TGFB-induced factor homeobox 1 [Source:RGD Symbol;Acc:1310517]
Thbs1	thrombospondin 1 [Source:RGD Symbol;Acc:1588455]
Thbs2	thrombospondin 2 [Source:RGD Symbol;Acc:1310979]
Cebpb	CCAAT/enhancer binding protein beta [Source:RGD Symbol;Acc:2327]
Jun	Jun proto-oncogene, AP-1 transcription factor subunit [Source:RGD Symbol;Acc:2943]
Myc	MYC proto-oncogene, bHLH transcription factor [Source:RGD Symbol;Acc:3130]
Nfkb1	nuclear factor kappa B subunit 1 [Source:RGD Symbol;Acc:70498]
Sp1	Sp1 transcription factor [Source:RGD Symbol;Acc:3738]
Stat1	signal transducer and activator of transcription 1 [Source:RGD Symbol;Acc:1305747]
Stat6	signal transducer and activator of transcription 6 [Source:RGD Symbol;Acc:1309063]
Akt1	AKT serine/threonine kinase 1 [Source:RGD Symbol;Acc:2081]
Bcl2	BCL2, apoptosis regulator
Faslg	Fas ligand [Source:RGD Symbol;Acc:3880]
