# Supplementary figures and images for: Molecular changes, histopathology, and ultrasonic vocalization acoustic profiles of systemically dehydrated rats
Source: PLoS One. 2025 Apr 22;20(4):e0322187. doi: 10.1371/journal.pone.0322187 (PMC12013907; doi:10.1371/journal.pone.0322187)

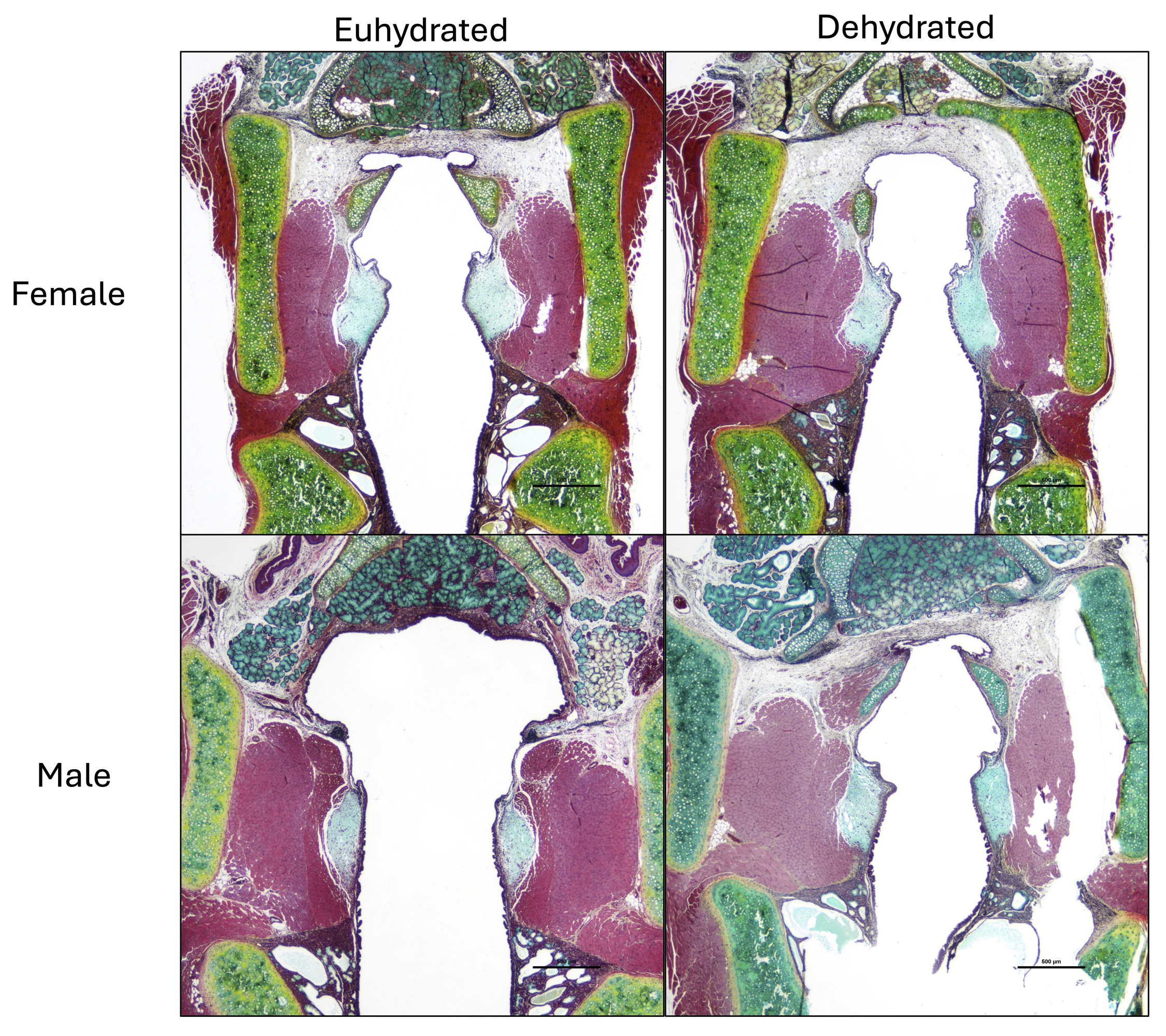

Supplement: S2 Fig — Images of pentachrome stained coronal larynx slices. The top two panels show example images from female larynges, and the bottom two panels show images from male larynges. The panels on the left side represent images from the euhydrated group, and the panels on the right side represent images from the dehydrated group. The colors of the stain correspond to the following tissue types: black – nuclei and elastic fibers, yellow – collagen fibers, blue – mucins, bright red – fibrins, and red – muscle fibers. (TIF) [file pone.0322187.s002.tif]

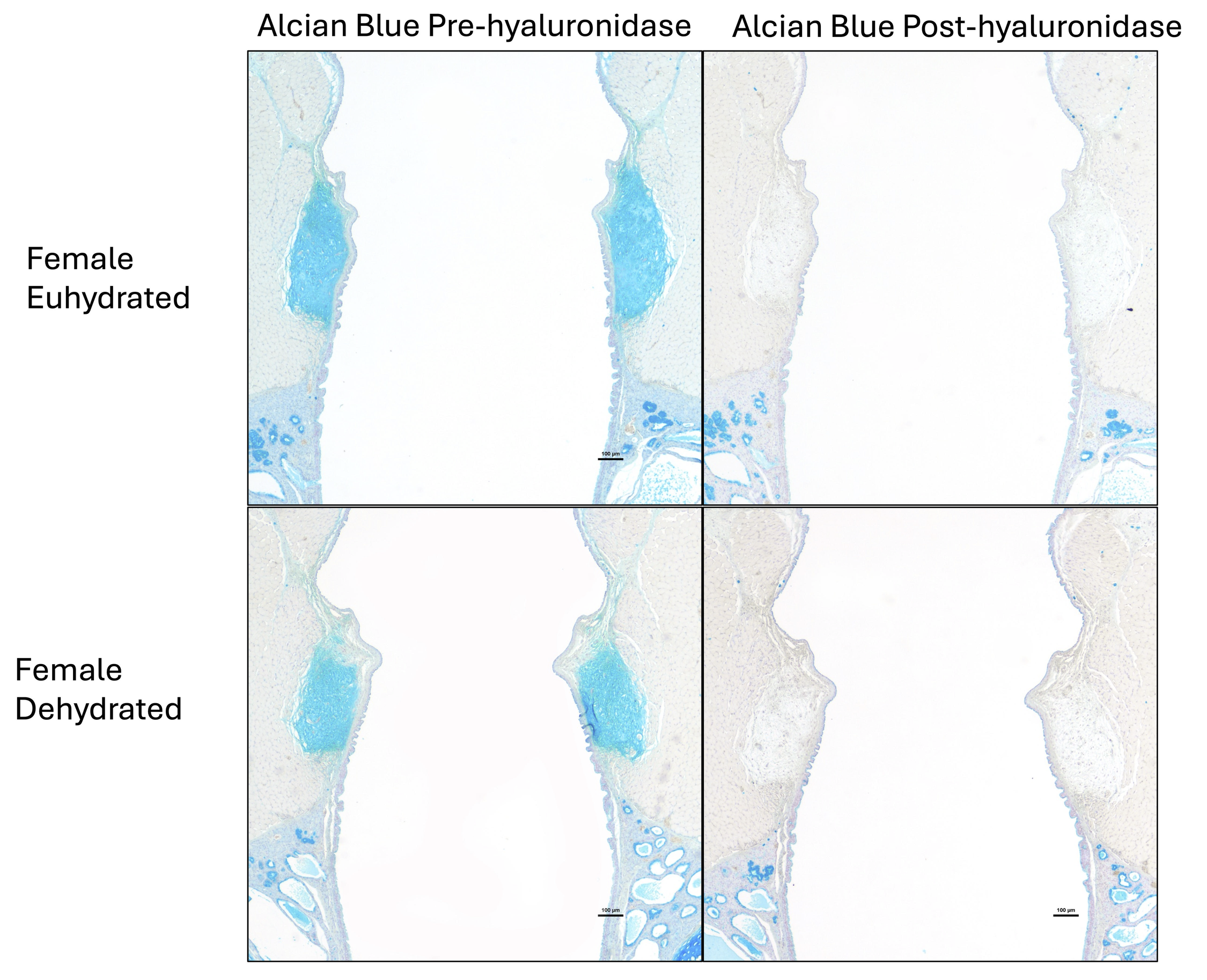

Supplement: S3 Fig — Images of Alcian blue stained coronal larynx slices from female rats before and after hyaluronidase treatment. The top two panels show example images of larynx slices from a female rat in the euhydrated group, and the bottom two panels show images of larynx slices from a female rat in the dehydrated group. The panels on the right side show the larynx slices before hyaluronidase treatment, and the panels on the right show the same larynx slices after treatment. (TIF) [file pone.0322187.s003.tif]

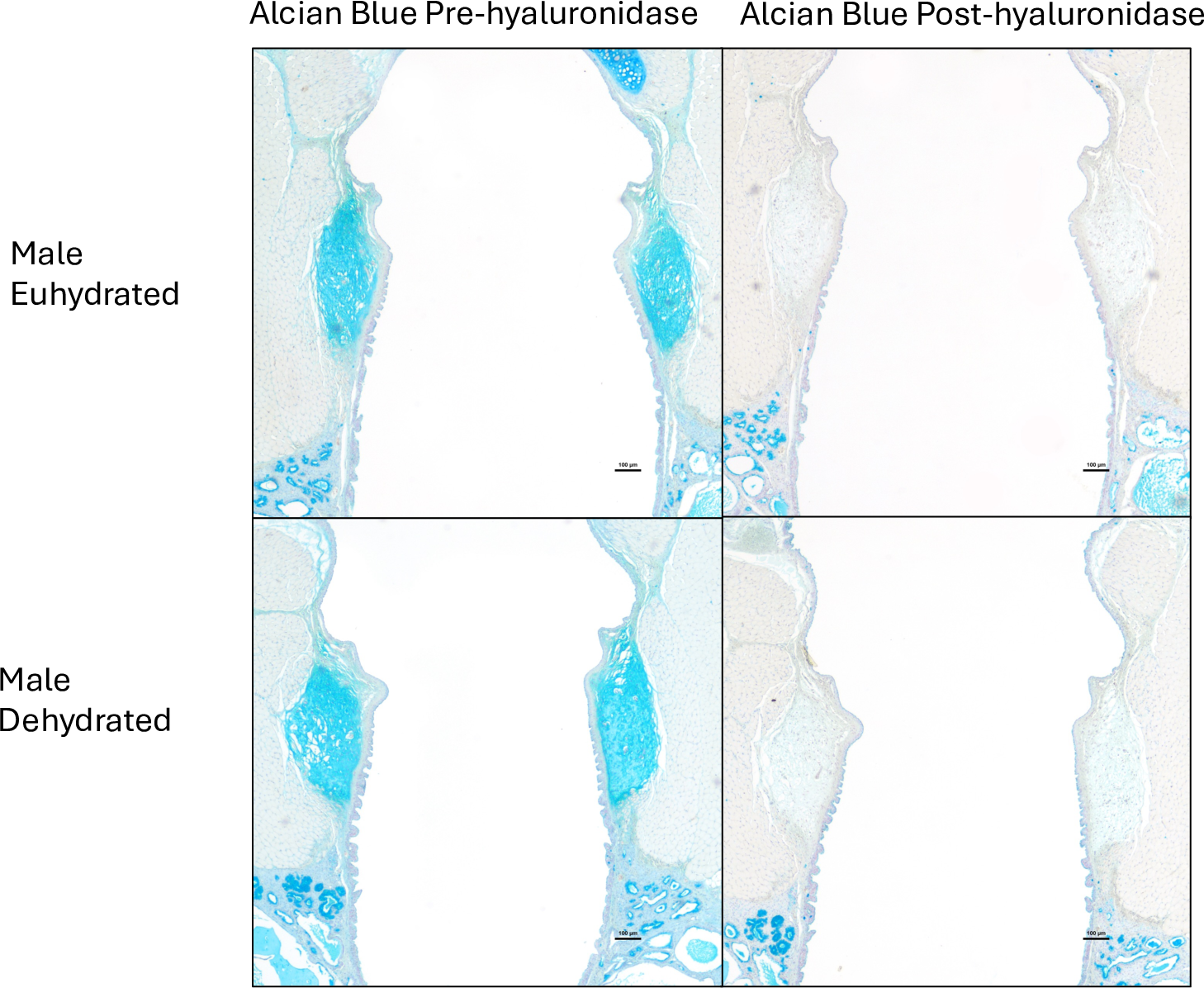

Supplement: S4 Fig — Images of Alcian blue stained coronal larynx slices from male rats before and after hyaluronidase treatment. The top two panels show example images of larynx slices from a male rat in the euhydrated group, and the bottom two panels show images of larynx slices from a male rat in the dehydrated group. The panels on the right side show the larynx slices after staining, and the panels on the right show the same larynx slices after hyaluronidase treatment. (TIF) [file pone.0322187.s004.tif]
